# Effects of *Mentha suaveolens* Essential Oil Alone or in Combination with Other Drugs in *Candida albicans*

**DOI:** 10.1155/2014/125904

**Published:** 2014-02-27

**Authors:** Annarita Stringaro, Elisabetta Vavala, Marisa Colone, Federico Pepi, Giuseppina Mignogna, Stefania Garzoli, Serena Cecchetti, Rino Ragno, Letizia Angiolella

**Affiliations:** ^1^Department of Technology and Health, Italian National Institute of Health, Viale Regina Elena 299, Piazzale Aldo Moro, 00161 Rome, Italy; ^2^Department of Public Health and Infectious Diseases, University of Rome “Sapienza”, Piazzale Aldo Moro, 00161 Rome, Italy; ^3^Department of Drugs Chemistry and Technology, University of Rome “Sapienza”, Piazzale Aldo Moro, 00161 Rome, Italy; ^4^Department of Biochemical Sciences “Rossi Fanelli”, University of Rome “Sapienza”, Italy; ^5^Department of Cell Biology and Neurosciences, Italian National Institute of Health, Viale Regina Elena 299, 00161 Rome, Italy

## Abstract

Candidosis is the most important cause of fungal infections in humans. The yeast *Candida albicans* can form biofilms, and it is known that microbial biofilms play an important role in human diseases and are very difficult to treat. The prolonged treatment with drugs has often resulted in failure and resistance. Due to the emergence of multidrug resistance, alternatives to conventional antimicrobial therapy are needed. This study aims to analyse the effects induced by essential oil of *Mentha suaveolens* Ehrh (EOMS) on *Candida albicans* and its potential synergism when used in combination with conventional drugs. Morphological differences between control and EOMS treated yeast cells or biofilms were observed by scanning electron microscopy and transmission electron microscopy (SEM and TEM resp.,). In order to reveal the presence of cell cycle alterations, flow cytometry analysis was carried out as well. The synergic action of EOMS was studied with the checkerboard method, and the cellular damage induced by different treatments was analysed by TEM. The results obtained have demonstrated both the effects of EOMS on *C. albicans* yeast cells and biofilms and the synergism of EOMS when used in combination with conventional antifungal drugs as fluconazole (FLC) and micafungin (MCFG), and therefore we can hypothesize on its potential use in therapy. Further studies are necessary to know its mechanism of action.

## 1. Introduction

The yeast *Candida albicans* is the most important cause of fungal infections in humans, especially in immunocompromised patients [1, 2].

Microbial biofilms play an important role in human diseases. It is now estimated that 65% of infections are caused by microorganisms growing on surfaces rather than in the free living planktonic state [3, 4]. Biofilms are structured microbial communities attached to surface and encased within a matrix of exopolymeric substances. Candida biofilms are difficult to eradicate because of their very high antifungal resistance to the common antifungal [5]. The prolonged treatment of *C. albicans *with conventional drugs has determined frequent therapeutic failures.

Essential oils or and some of their constituents have been indeed shown to be effective against a large variety of organisms including bacteria [6–8], virus [9], fungi [10–13], protozoa, and parasites [14, 15]. Many essential oils are liquid, volatile, limpid and rarely colored, lipid soluble, and soluble in organic solvents with a generally lower density compared to water [16]. It was proved that essential oils can damage the eukaryotic cells with prooxidant effect on the cell membrane and on some organelles, such as mitochondria. In eukaryotic cells the change in mitochondrial membrane permeability leads to cell death by apoptosis and necrosis [17]. Moreover, EOs have shown prooxidants actions on proteins and DNA with the production of reactive oxygen species (ROS). These may cause damage to cellular macromolecules and in some cases induce the formation of covalent adducts to DNA, proteins, and membrane lipids [17].

According to the literature, the investigation of natural products active against *C. albicans* increased significantly in the last 10 years, with the investigation of approximately 258 plant species from 94 families [18].

The concept of antimicrobial synergy is based on the principle that, in combination with other drugs, the formulation may enhance efficacy, reduce toxicity, decrease adverse side effects, increase bioavailability, lower the dose, and reduce the development of antimicrobial resistance [19, 20]. The research of new antimicrobial protocols which include natural products has recently increased. For these reasons, our interest was directed to the study of antifungal effects of *Mentha suaveolens* essential oil, alone or in combination with other antifungal drugs.

In a previous work [21], we demonstrated “*in vitro”* and “*in vivo” Mentha suaveolens* essential oils (EOMS) antifungal activity against different *C. albicans *strains sensitive and resistant to synthetic drugs and other fungi [22]. All strains of *C. albicans* tested against EOMS, both sensitive and resistant to synthetic drugs, showed the same MIC and MFC values.

In the present paper, the aim of our study has been to establish the effects of EOMS on both biofilm formation and cell cycle progression of *C. albicans* strain CO23. Moreover, we have evaluated antifungal activity and synergistic effects of EOMS, with fluconazole and micafungin by NCCLS microdilution method, associated with the broth microdilution checkerboard assay.

## 2. Materials and Methods

### 2.1. Extraction, Gas Chromatography, and Mass Spectroscopy Analysis

The essential oil of *Mentha suaveolens* (EOMS patent WO 2011092655 A3) was extracted using a four-hour hydrodistillation of the leaves using a Clevenger-type apparatus. The chemical composition of EOMS was described previously [22–24]. The EOMS major component was found to be piperitenone oxide (PEO) at about 90%.

### 2.2. Organisms and Growth Conditions

The CO23 strain of *C. albicans* was isolated from a subject with vulvovaginal candidiasis, susceptible to micafungin (MCFG, MIC 0.025 *μ*g/mL) and fluconazole (FLC MIC 0.25 *μ*g/mL). The strain was used throughout the study and was routinely maintained on Sabouraud dextrose agar medium (SDA; Difco, Detroit, MI) at 28°C.

### 2.3. Adherence Assay and Biofilm Formation

In order to examine the effect of the EOMS on the adherence and biofilm formation, the adherence cells were grown for 24 h at 28°C in YPD broth, washed twice with sterile PBS (10 mM phosphate buffer, 2.7 mM potassium chloride, 137 mM sodium chloride, pH 7.4), and resuspended at 37°C in RPMI 1640 supplemented with morpholinepropanesulfonic acid (MOPS) plus 10% FBS at 1.5 × 10^3^ cells/mL. After incubation for 3 h at 37°C in six-well polystyrene plates (Corning Incorporated, Corning, NY) followed by extensive washing, 1 mL of Sabouraud dextrose agar was poured into each well and let it solidify. After incubation at 37°C for 24 h, colonies were counted, and the results were expressed as a percentage of the inoculum size. The inoculum size for each cell suspension was confirmed by plating aliquots of the culture directly in Sabouraud dextrose agar plates.

For biofilm formation, cells grown as described above were seeded at a density of 1 × 10^6^ cells/mL in presterilized, polystyrene, flat-bottom six-well microtiter plates (Corning, NY) and incubated for 24 h at 37°C. After biofilm formation, the medium was aspirated, and nonadherent cells were removed by thoroughly washing the biofilm three times with sterile PBS. All the experiments were carried out in the presence or in absence of EOMS (concentration ranging from 0.78 to 1.56 mg/mL) diluted in RPMI-1640 supplemented with Tween 80 (final concentration of 0.001% *v*/*v*). A semiquantitative measurement of metabolic activity of biofilm formation was made by CV assay [25, 26], the biofilms were fixed with 99% *v*/*v* methanol for 15 minutes, and then 0.02% (*v*/*v*) CV was added for 20 minutes. Biofilms were washed and 33% (*v*/*v*) acetic acid was added again for 30 minutes. The CV released was measured at 590 nm. All biofilm and control assays were carried out three times in triplicate.

### 2.4. Scanning Electron Microscopy (SEM)

For SEM, *C. albicans* cells were grown in glucose supplemented YNB medium (see above) with or without 0.33 mg/mL EOMS at 28°C for 24 h. At the same time, the cells were grown in the presence of both EOMS and drugs. After washing twice in calcium and magnesium-free phosphate-buffered saline (PBS), the cellular pellets resulting from centrifugation were fixed for 20 min at room temperature with 2.5% (*v*/*v*) glutaraldehyde in 0.01 M cacodylate buffer (pH 7.4) containing 2% (*w*/*v*) sucrose. After 3 washes in the same buffer, the cells were postfixed with 1% (*w*/*v*) OsO_4_ for 1 h, dehydrated on an ethanol gradient, critical point dried in CO_2,_ and gold coated by sputtering. The samples were examined with a Cambridge Stereoscan 360 scanning electron microscope (Cambridge Instruments, Cambridge, UK).

For analysis by transmission electron microscopy (TEM), cells were prefixed with glutaraldehyde, as above, and then postfixed with the OsO_4_ solution overnight, at 4°C. The cells were then dehydrated in acetone gradient and embedded in epoxy resin (Agar 100 resin, Agar Scientific Ltd., Stansted, UK) as per routine procedure [27]. Ultrathin sections, obtained with LKB Ultrotome Nova, were stained with uranyl acetate and lead citrate and examined with Philips 208 transmission electron microscope (FEI Company, Eindhoven, The Netherlands).

### 2.5. Cell Cycle Analysis

For cell cycle analysis by flow cytometry, the pellet of untreated and treated samples was fixed in 70% ethanol in PBS at 4°C for 7 days, washed twice with cold PBS, and then resuspended in PBS containing PI (40 *μ*g/mL final concentration) and 100 *μ*g/mL RNAse at 37°C for 30 min. Samples were then analysed with cytometry, using a FACScan flow cytometer.

All fluorescences were analysed with a FACScan flow cytometer (Becton, Dickinson & Company, Franklin Lakes, NJ, USA) equipped with a 15 mW, 488 nm, and an air-cooler argon ion laser. The fluorescent emissions were collected through a 575 nm band-pass filter for PI and acquired in linear mode. At least 10,000 events were analysed.

### 2.6. Minimal Inhibitory Concentration (MIC)

The Minimal Inhibitory concentration (MIC) was determined by microbroth dilution method (microsterile plate) according to the Clinical and Laboratory Standards Institute/National Committee for Clinical Laboratory Standards (CLSI/NCCLS) Approved Standard M27-A3, 2008 [28]. The minimal inhibitory concentration (MIC) was determined as the lowest concentration of drugs or essential oils at which no microbial growth was observed. Fluconazole (FLC) and micafungin (MCFG) 0.5 mg/mL solution were prepared dissolving the agent in endotoxin free water. Solutions of essential oil (100 mg/mL) were prepared in RPMI 1640. Briefly, to determine the MIC of EOMS, or FLC, and MCFG, RPMI-1640 supplemented with MOPS at pH 7 was used. EOMS was diluted in RPMI-1640 supplemented with Tween 80 (final concentration of 0.001% *v*/*v*). The dilution, ranging from 0.01219 to 12.48 mg/mL of the essential oil, was prepared in 96 well plates. The inoculum size was about 2.5 × 10^3^ cells/mL. The plates were incubated at 30°C for 24–48 h.

### 2.7. Checkerboard Method Used to Evaluate the Synergic Action of EOMS with Conventional Drugs

Twelve serial twofold dilutions of EOMS were prepared following the same broth dilution method adopted to assess MICs. A dilution of the oil was prepared (0.01219 to 12.48 mg/mL), together with a series of two fold serial dilutions of FLC in the 0.06–64 *μ*g/mL range and MCFG in the range (0.03–8 *μ*g/mL). In this way, all FLC and MCFG dilutions were mixed with the appropriate concentration of oil, thus obtaining a series of different concentrations [29]. The analysis of the combination of the substances was carried out calculating the fractional inhibitory concentration (FIC) index. The FIC index (FICI) was calculated dividing the MIC of the combination of EO and the reference antifungal by the MIC of EO or reference antifungal alone:

(1) FIC of oil =MIC of oil in combination with antifungal drugsMIC of oil alone,FIC of antifungal drug =MIC of antifungal in combination with oilMIC of antifungal drug,FICI=FIC of oil+FIC of antifungal drug.

The FICI, obtained by adding both FICs, was interpreted as indicating a synergistic effect when it was ≤0.5, as additive or indifferent when it was >0.5 and ≤2, and as antagonistic when it was >2 [29].

## 3. Results

### 3.1. Adherence and Biofilm Formation

**Figure 1:**
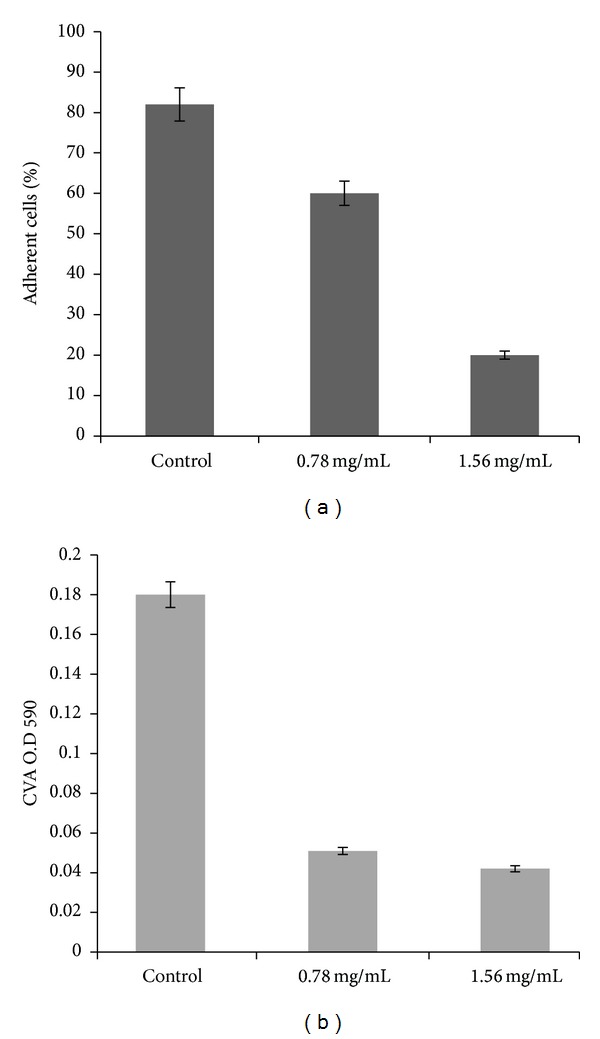
Adherence assay and biofilm formation. After incubation at 37°C for 24 h with EOMS, colonies were counted, and the results were expressed as percentage of adherence cells (a). In the biofilm formation (b), after incubation at 37°C for 24 h, cells were tested by CV assay (absorbance 590 nm). The values represent the results of three experiments.

In previous studies [21], the effect on preformed hyphae was examined after 24 h of treatment with EOMS. A concentration of 0.5 mg/mL was able to inhibit partially the hyphal form (about 50%). As hyphal formation is essential to the structural integrity of biofilm, additional experiments were carried out to assess the capacity of *C. albicans* to adhere to the substrate and the biofilm formation in the presence or in the absence of EOMS (0.78 and 1.56 mg/mL). As shown in Figure 1, in the presence of EOMS, the adhesion and biofilm formation were significantly decreased at both concentrations (Figures 1(a) and 1(b)). Indeed, the percentage of adherent cells decreased from 20% to 60% at the concentration of 0.78 mg/mL and 1.56 mg/mL, respectively (Figure 1(a)). This result was also confirmed with CV assay that characterizes the metabolic activity of the biofilm. In the presence of EOMS, there was a high reduction of metabolic activity: about 80% compared to control (Figure 1(b)).

### 3.2. SEM Observations

**Figure 2:**
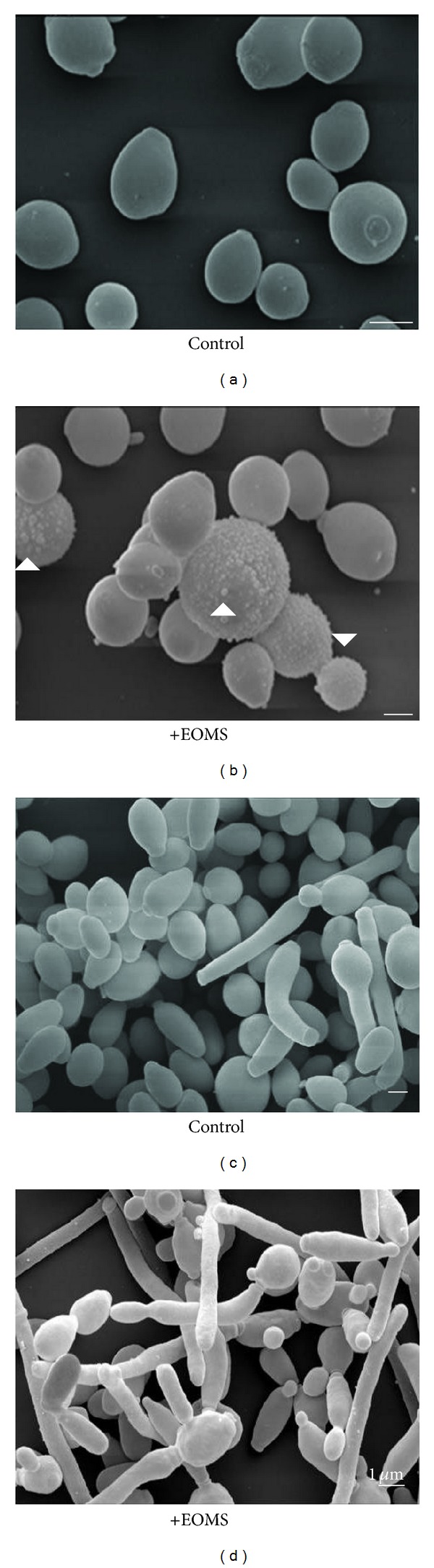
SEM images of yeasts and biofilms. (a) Untreated yeastcells; (b) yeast cells treated with 0.33 mg/mL EOMS; (c) untreated *Candida* biofilm; (d) *Candida* biofilm treated with 0.33 mg/mL EOMS.

In order to examine morphological alterations induced by EOMS on *C. albicans,* yeast cells, and biofilm formation, cells were treated at sub-inhibitory concentration of 0.33 mg/mL of EOMS and analyzed by SEM. Figures 2(a) and 2(c) show untreated *C. albicans* cells and *Candida *biofilm formation, respectively.  Figure 2(a) shows that untreated *C. albicans* cells had their typical shape, dimension, and surface morphology. When these cells were treated with EOMS at sub-lethal concentration of 0.33 mg/mL, evident morphological alterations “bubbles” were observed on the cell wall (Figure 2(b), arrowheads). In addition, some cells were swollen and stretching to form clusters.  Figure 2(c) shows *Candida* biofilm as a mixture of blastospore, pseudohyphae, and hyphae. After EOMS treatment, SEM analysis also reveals modifications of *Candida* biofilm: the biofilm structure appears to be reduced and morphological alterations are visible on hyphae cells (Figure 2(d)).

### 3.3. Confocal Observations and Cell Cycle Analysis by FACS

**Figure 3:**
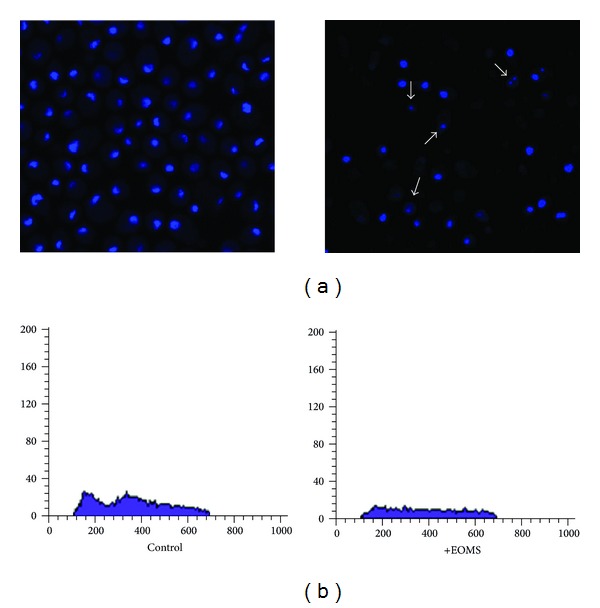
Laser scanning confocal microscopy after Hoechst staining and cell cycle analysis by FACS. *C. albicans* cells untreated or treated with EOMS were observed by a LSCM after Hoechst staining (a) and analysis by FACS of cell cycle (b).

Hoechst staining observations by laser scanning confocal microscopy (LSCM) of cells treated with EOMS for 3 h revealed chromatin condensation with initial nuclear shrinkage without nuclear fragmentation in treated yeast cells (Figure 3(a), right panel, arrows) while untreated yeast cells did not show these alterations (changes). FACS analysis of cell cycle showed that a sub-lethal dose of EOMS (0.33 mg/mL) after 3 h of the treatment induced slow-down of cell growth compared to untreated control cells (Figure 3(b), right and left panel, resp.).

### 3.4. The Fractional Inhibitory Concentration Index (FICI)

To explore the possibility of developing a more powerful combination therapy of *Mentha suaveolens* essential oil with fluconazole or micafungin, the checkerboard microtiter test was performed.  Table 1 shows the results obtained in terms of the minimal inhibitory concentration (MIC) EOMS inhibition of the growth of *C. albicans* was achieved at 0.78 mg/mL, while the MIC of antifungal drugs of FLC and MCFG were 2 and 0.0156 (mg/mL) respectively.

The FICIs calculated from the results of the checkerboard titer assays (Table 1) revealed the following: treating *C. albicans* with fluconazole in combination with EOMS caused a significant decrease in the MIC, compared to their individual MIC values. For example, the MIC of fluconazole alone against *C. albicans* was lowered from 2 to 0.5 (*μ*g/mL) in the presence of EOMS. The MIC of EOMS alone also decreased from 0.78 to 0.095 (mg/mL). Synergistic effects were obtained using various combinations of EOMS with MCFG and FLC with FICIs of 0.53 and 0.37, respectively. Nevertheless, the results indicate that the synergistic effect of EOMS with FLC was stronger than a combination with MCFG.

### 3.5. TEM Observations

**Figure 4:**
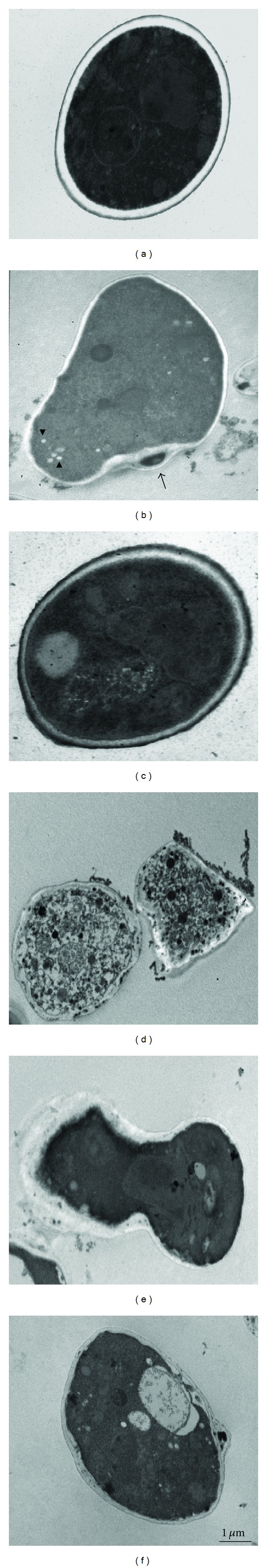
TEM observations on *C. albicans* cells treated alone or in combination with EOMS and antifungal drugs. (a) Untreated yeast cells; (b) yeast cells treated with EOMS alone; (c) yeast cells treated with FLC alone; (d) yeast cells treated in combination with EOMS and FLC; (e) yeast cells treated with MCFG alone; (f) yeast cells treated in combination with EOMS and MCFG.

In order to evaluate the possible cytological damages caused by synergism of EOMS and drugs, TEM observations were performed.  Figure 4 shows untreated cells (*C. albicans*) (Figure 4(a)), a cell treated with EOMS (Figure 4(b)), FLC (Figure 4(c)), and MCFG (Figure 4(e)) alone, or a combination of EOMS plus FLC (Figure 4(d)) or MCFG (Figure 4(f)). Minimal ultrastructural alterations were observed in cells treated with EOMS alone or with synthetic drugs alone (FLC and MCFG, resp.). Even so, TEM analysis revealed some alterations in the cell walls morphology. Moreover, significant ultrastructural modifications were observed in response to combined treatments (Figures 4(d) and 4(f)). Actually, alterations were evident both in the cytoplasm and in the cell wall. The complete destruction of some cells were caused by the combined treatment, overall after treatment with EOMS and FLC combination (Figure 4(d)). Probably, this was due to their synergistic activity on the yeast membranes.

## 4. Discussion

Adherence is an important step in biofilm formation, and biofilm formation by *Candida albicans* causes resistance to antifungal drugs and thus is of major clinical relevance. Antifungal drug resistance in fungal biofilms is both complex and multifactorial. The different factors involved are cell density, stress, persistence, extra cellular matrix, efflux, overexpressed targets, and the general physiology of the biofilm [5].

During the last decade, a variety of essential oils have been screened to assess their antimicrobial activity as potential sources of new antimicrobial compounds and as alternatives drugs to treat infectious diseases and/or to promote food preservation. Different components of essentials oils vary among plants species and parts; most of these components are derived from terpenes and their oxygenated derivatives, that is, terpenoids, that are aromatic and aliphatic acid esters and phenolic compounds [30]. While essential oils were extensively tested against a broad spectrum of bacteria, yeast and fungi [31], the mechanism of the inhibitory action of essential oil on the microbes is not well understood. As yet, there are a limited number of studies describing the mode of action of many essential oils.

The antimicrobial activity of the essential oils can be explained by the lipophilic character of the monoterpenes contained in them. The monoterpenes act by disrupting microbial cytoplasmic membrane, which thus loses its high impermeability for protons and bigger ions; if disturbance of membrane integrity occurs, then its functions are compromised not only as a barrier but also as a matrix for enzymes and as an energy transducer [32]. However, specific mechanisms involved in the antimicrobial action of monoterpenes remain poorly characterized.

For example, the antibacterial properties of tea-tree oil (TTO) have been attributed to the monoterpenoid, terpinen-4-ol, and since monoterpenoids are lipophilic, it is thought to diffuse into and damage cell membrane structures, causing increased fluidity or disordering membrane structure and inhibition of membrane-bound enzymes [33].

Moreover, Giordani et al. [34] demonstrated the perturbation of molecular architecture of the plasma membrane of human cancer cell lines, without completely disrupting it, using TTO and terpinen-4-ol. Accordingly, the antimicrobic effect of terpinen4-ol may be due to perturbation of the lipidic fraction of microorganism plasmic membrane, resulting in alterations of membrane properties. Thus, the monoterpenes might cross the cell wall and the cell membranes of the yeast cells, interacting with intracellular sites, key points for *Candida* activity [35].

Hammer et al. [36] investigated the antifungal effect of (TTO) and several of its components on different yeasts. Other authors have demonstrated that essential oils not only possess antimicrobial activity against bacteria and fungi existing in planktonic and biofilm modes of growth [37], but also show synergistic activity when combined with other drugs effective on biofilms of different microorganisms [38].

Previously we demonstrated an antimycotic activity “*in vitro”* and “*in vivo”* of *Mentha suaveolens* essential oil (EOMS) against dermatophytes such as the yeasts *C. albicans *and Cryptococcus spp.[21, 22]. *Mentha suaveolens* plants collected in various regions of Morocco contain a high percentage of monoterpenes oxides, such as piperitenone oxide and piperitoneoxide, terpenic alcohol (phenol, p-cymen-8-ol, geraniol, terpineol, and borneol) and terpenic ketones (pulegone and piperitenone); all of them account for 65% to 90% of the total essential oil.

In particular, all strains of *C. albicans* tested, both sensitive and resistant to synthetic drugs, showed the same MIC and MFC values ranging from 0.78 to 1.56 mg/mL, confirming the antimycotic activity of this essential oil, as was also reported by other authors [23, 24].

In this study, we have evaluated the adherence and the metabolic activity by CV assay in the presence or in the absence of EOMS in a sensitive strain of *C. albicans* and the effects of EOMS on biofilm formation, cell cycle progression, and its antifungal activity, alone or in combination with other synthetic or conventional drugs. Our results (Figures 1(a) and 1(b)) clearly show a decrease of adherence and metabolic activity in the presence of different concentrations of EOMS, with a decrease of about 40% in adherence and about 70–80% of inhibition of biofilm formation. The inhibition of biofilm formation has also confirmed by SEM observations (Figure 2(d)) in also, the inhibitory effect of EOMS on biofilm formation was confirmed by SEM observations (Figure 2(d)).

EOMS treatment on CO23 yeast cells was able to induce morphological surface modifications of cell wall as revealed by SEM observations (see bubbles, Figure 2(b)) and in some cases, cells were swollen and forming clusters. EOMS induced also modifications of the CO23 cell cycle as revealed by FACS analysis. In addition, EOMS cytotoxic effects were also investigated by LSCM and TEM observations. Actually, our results confirm that EOMS, primarily monoterpenes oxides, was also able to induce both the inhibition of the correct synthesis of the cell wall (Figure 4(b), arrows) and rarefied cytoplasmic matrix with the presence of some small electron-transparent vacuoles (Figure 4(b), arrowheads) with slow-down of yeast cell growth (reduction of Phase S) (see Figure 3(b), right panel) probably due to chromatin condensation as observed by LSCM (Figure 3(a), right panel, arrows).

As known, the azoles, particularly FLC which has as target the ergosterol biosynthesis pathway, a lipid present in fungal membranes, remain among the most common antifungal drugs, but FLC-intensive clinical use for both therapy and prophylaxis has favored the emergence of resistant strains. The threat of increasing resistance to azole drugs, associated with the relative scarcity of antifungal drugs, prompted the development of new drugs such as the echinocandins (e.g., MCFG). These cytocidal drugs inhibit cell wall synthesis through the inhibition of *β*-1,3 glucan synthase and have rapidly become an important therapeutic option for several fungal infections. Recent research work has demonstrated that synergism of natural products and antibiotics is a thrust area of phytomedicinal research and for developing new prospectives for phytopharmaceuticals [36, 37]. New antimicrobial combinations of drugs including natural products have recently become a priority of research. This approach has financial implications as reformulation of existing drugs or combinations may prove to be a better option than developing a new chemical drugs that require extensive testing and preclinical trials. The synergism of plant derived compounds and antibiotics has been found to be very effective against infectious diseases [38] and also against *C. albicans* [39]. A clear synergistic effect of essential oils with antifungal agent amphotericin B has been reported against *C. albicans *infections [40].

The first aim of this study was to assess the antimicrobial effects of EOMS against *C. albicans* cells, in particular against their biofilm growth. Secondly, we sought to evaluate the effect of combining these essential oil and synthetic drugs (FLC and MCFG) with a view to reduce the concentration of FLC and MCFG necessary to achieve a particular level of growth inhibition against *Candida*.

Our results indicate that using various combinations of EOMS with FLC and MCFG was observed synergistic effects. The combination of EOMS with FLC resulted stronger than the combination of EOMS with MCFG (FICI of 0.37 and 0.53, resp.).

Our hypothesis is that EOMS monoterpenes, after their passage across the cell wall, damage the lipid bilayer of the cell membrane increasing the *Candida* cell membranes permeability and promoting FLC action on ergosterol leading to the cell damage. Further demonstration of the existence of synergism between EOMS and conventional antifungal drugs (FLC and MCFG) was obtained by TEM observations (Figure 4), where the complete destruction of cytoplasmatic material of *Candida* cells (probably the induction of cell death) became evident in Figure 4(d). On the other hand ( Figure 4(f)), the combination of EOMS and MCFG (additive effect) appeared less effective than EOMS and FLU (synergic effect) as also demonstrated by its FICI values.

In conclusion, this study shows for the first time that (1) EOMS is able to induce the morphological alterations on the cell wall and cell membranes of *Candida* cells with perturbation of the cell cycle associated with nuclear condensation and (2) the efficiency of treatment of EOMS and FLC in combination is more than additive, that is, synergic against *C. albicans* cells.

Such combined treatment may reduce the doses of the conventional drugs such as FLC and MCFG used and minimize the side effects. In addition, the use of EOMS might also be able to counteract the development of fungal resistance against such established drugs which increasingly give problems in clinical practice, when using common antifungal therapeutics. Of course, further experiments will be necessary to assess the potential of such combined treatments (EOMS with antifungal drugs) in view of possible therapeutic application.

## Figures and Tables

**Table 1:** Fractional inhibitory concentrations (FIC) and indices (FICIs) of antifungal drugs fluconazole (FLC) and micafungin (MCFG) combined with the essential oil of *Mentha suaveolens* against *Candida albicans*.

	MIC_a_	MIC_c_	FIC	FICI
EOMS-fluconazole				
* M. suaveolens* (mg/mL)	0.78	0.095	0.25	0.375
Fluconazole (*μ*g/mL)	2	0.5	0.125	
EOMS-micafungin				
*M. suaveolens* (mg/mL)	0.78	0.0243	0.031	0.531
Micafungin (*μ*g/mL)	0.0312	0.0156	0.5	

MIC_a_: MIC of the sample alone; MIC_c_: MIC of the sample of the most effective combination. FIC of oil = MIC of oil in combination with antifungal drugs/MIC of oil alone. FIC of antifungal drug = MIC of antifungal drugs in combination with oil/MIC of antifungal drugs. FICI = FIC oil + FIC of antifungal drug.
